# Unravelling the role of nanomedicine in attenuating inflammation, oxidative stress and cellular ageing in chronic obstructive pulmonary disease

**DOI:** 10.1007/s00204-025-04116-x

**Published:** 2025-06-29

**Authors:** Lokesh Nagar, Annu Saini, Sukriti Vishwas, Sachin Kumar Singh, Gaurav Gupta, Ronan MacLoughlin, Raimar Lobenberg, Neal M. Davies, Dinesh Kumar Chellappan, Keshav Raj Paudel, Kamal Dua, Harish Dureja

**Affiliations:** 1 Department of Pharmaceutical Sciences, Maharshi Dayanand University, Rohtak, 124001 India; 2 Chitkara College of Pharmacy, Chitkara University, Rajpura, Punjab 140401 India; 3 School of Medical and Life Sciences, Sunway University, Kuala Lumpur, Malaysia; 4 Faculty of Health, Australian Research Centre in Complementary and Integrative Medicine, University of Technology, Sydney, Ultim, NSW 2007 Australia; 5 Centre of Medical and Bio-Allied Health Sciences Research, Ajman University, Ajman, United Arab Emirates; 6 Research and Development, Science and Emerging Technologies, Aerogen Limited, IDA Business Park, Dangan, Galway, H91 HE94 Ireland; 7 School of Pharmacy & Biomolecular Sciences, Royal College of Surgeons in Ireland, Dublin, D02 YN77 Ireland; 8 School of Pharmacy & Pharmaceutical Sciences, Trinity College, Dublin, D02 PN40 Ireland; 9 Faculty of Pharmacy and Pharmaceutical Sciences, University of Alberta, Edmonton, AB T6G 2P5 Canada; 10 Department of Life Sciences, School of Pharmacy, IMU University, Bukit Jalil, 57000 Kuala Lumpur, Malaysia; 11 Centre for Inflammation, Centenary Institute and University of Technology Sydney, School of Life Sciences, Faculty of Science, Sydney, NSW 2007 Australia; 12 Woolcock Institute of Medical Research, Macquarie University, Sydney, NSW Australia; 13 Uttaranchal Institute of Pharmaceutical Sciences, Uttaranchal University, Dehradun, India; 14 Discipline of Pharmacy, Graduate School of Health, University of Technology, Sydney, Australia; 15 NICM Health Research Institute, Western Sydney University, Westmead, NSW 2145 Australia; 16 School of Pharmacy, Graphic Era (Deemed to be University), Clement Town, Dehradun, 248002 India

**Keywords:** COPD, Cellular senescence, Nanoparticles, Nanotubes, Oxidative stress

## Abstract

Chronic obstructive pulmonary disease is a chronic lung disease which causes obstruction and inflammation in the airways or other parts of the lung. It is often associated with structural changes in the lung due to persistent inflammation caused by prolong exposure to cigarette smoke. Other factors such as oxidative stress, chronic inflammation and cellular senescence also play a major role in the progression of the disease. Chronic inflammation is responsible for cell cycle dysfunction. Cellular senescence is associated in the pathogenesis of COPD, which can accelerate the lung aging process. Cellular senescence can elevate the level inflammatory mediators, which can comprise lung function and structure. This review explores various pathologic mechanisms which are involved in the progression of COPD. It also explores the application of nanostructure-based drug delivery systems such as solid lipid nanoparticles, polymeric nanoparticles, liposomes, nanoemulsions, dendrimers and other miscellaneous nanostructures in overcoming challenges associated with current conventional treatments for COPD. This review explores recent advancements in the field of nanostructures-based drug delivery systems for COPD treatment.

## Introduction

Chronic obstructive pulmonary disease is a chronic lung disease which causes obstruction and inflammation in the airways or other parts of the lung (World Health Organisation 2024). Coughing, breathing difficulties, wheezing, and fatigue are some of the symptom associated to COPD (Sarkar and Bhardwaz 2019). COPD is a highly prevalent condition that majorly contributes to several deaths globally (Czarnecka-Chrebelska et al. 2023). The World Health Organization (WHO) reported that COPD was the 3rd leading cause of death in 2019, with 3.23-million deaths in that year (World Health Organisation 2024). The data published by disability-adjusted life years (DALYs) stated that COPD is in the 7th rank in line of serious illnesses globally. Based on reports and cases, COPD can also be called as a luxury disease as the 70% COPD cases found only in high-income countries, where tobacco smoking is the main reason for this (Parums 2023). In low- and middle-income countries, household air pollution is mainly responsible for COPD, while tobacco smoking only contributes 30% of total COPD cases (Adeloye et al. 2022; Sana et al. 2018). These factors collectively provoke damage, inflammation, and structural changes in the airways and lung tissues (Aghasafari et al. 2018).

The aging is well-known with the words growing old, which involves continuous cellular changes in the organisms, which, if stay for long may lead to senescence (Guo et al. 2022). In scientific terms, a stage where cell permanently loses its capacity to divide, but remains alive is known as senescent cells (van Deursen 2014). As time goes on, a number of these senescent cells accumulate in various tissues throughout the body, which stay active and starts emitting harmful substances, which potentially leads to inflammation and damage to healthy cells all around (McHugh and Gil 2017; Chen et al. 2018). The accumulation and gradual increase in senescent cells further leads to tissue dysfunction, age-related illnesses, and a decrease in lifespan (Kumari and Jat 2021; Mijit et al. 2020; Childs et al. 2015). In patients with COPD, there is a notable accumulation of senescent cells within the lungs and the main cause is cigarette smoke (Barnes et al. 2019). Oxidative stress produced by cigarette smoke can trigger the senescence in COPD (Barnes 2022). The gathering of senescent cells in airway causes the progression of small airway fibrosis and loss of alveolar cells, which further leads to emphysema in COPD (Rivas et al. 2022). The oxidative stress produced by cigarette smoke elevated the level of ROS (reactive oxygen species) in the lung which further causes the low grade inflammation and shortened telomere, a sign of senescent or aging lung (Parikh et al. 2019; Bezerra et al. 2023; Nousis et al. 2023).

The development of COPD in the ageing population entails a series of molecular and cellular processes which includes different inflammatory mediators and biomarkers (Bailey et al. 2018). Generally, aged individuals with COPD use inhalants for the relief but prolonged use of inhalants can cause irritation which results in increasing oxidative stress and inflammation in the airway and lung tissues (Taucher et al. 2022). After inflammation in lungs, the inflammatory mediators like interleukin-6 (IL-6), tumor necrosis factor-alpha (TNF-α), and IL-8 elevated their level which ultimately cause tissue injury (Manosalva et al. 2023). This tissue injury process attracts and trigger immune cells such as neutrophils, macrophages, and T-lymphocytes, intensifying the immune response to damaged lung tissue, this gathering of cells restrict the airways and can cause breathing difficulties (Belchamber et al. 2021). In the lung of aged-COPD patients, the level of MDA (malondialdehyde) also increases with increase in oxidative stress, this elevation further leads to cellular damage and lungs dysfunction (Taniguchi et al. 2021). Moreover, the dysregulation in the equilibrium between metalloproteinases, and antiproteases, like alpha-1 antitrypsin can results in uncontrolled proteolytic processes (Cosio et al. 2016). This leads to the breakdown of lung elastin and collagen, which are also sign of COPD development (Mecham 2018).

## Molecular mechanisms contributing to aging/senescence in COPD

### Cigarette smoke

Cigarette smoke is a key factor for COPD development, which triggers cellular senescence by a well-known molecular pathway, which involves two different mechanisms (p53-p21 and p16INK4a-Rb) (Cha et al. 2023). These both mechanisms are fundamental regulators of cellular senescence (Takahashi et al. 2007). Exposure to cigarette smoke results in an increase of ROS and other toxic compounds which causes damage to DNA (Caliri et al. 2021). This process of DNA damage activates the DDR (DNA damage response), a key elevator of p53 protein (Steffens Reinhardt et al. 2023). This activation of p53 protein facilitates the expression of p21 protein, a CDK (cyclin-dependent kinase) inhibitor, which arrests the cell cycle, and causing cellular senescence (Al Bitar and Gali-Muhtasib 2019). Parallelly, cigarette smoke also elevates the expression of p16INK4a, which is also a CDK inhibitor. This p16INK4a hinders the function of Rb (retinoblastoma) protein, prompting cell cycle arrest (LaPak and Burd 2013). These above findings revealed the initiation of cellular senescence at a molecular level in COPD (Bateman et al. 2023). Cigarette smoke also disrupts anti-aging mechanisms by significantly reducing the expression of sirtuins, especially SIRT1 and SIRT3, in airway epithelial cells (Czarnecka-Chrebelska et al. 2023). The decrease in SIRT1 impairs its ability to deacetylate and stabilize FOXO3, a key transcription factor involved in cellular stress responses, thereby promoting premature cellular senescence and emphysema development (Gharipour et al. 2021). Similarly, reduced SIRT3 levels compromise mitochondrial function by lowering the expression and activity of manganese superoxide dismutase (MnSOD), a critical mitochondrial antioxidant enzyme (Adeloye 2022). This exacerbates mitochondrial oxidative stress, contributing to airway epithelial damage and COPD progression (Bezerra et al. 2023). Overall, cigarette smoke–induced downregulation of these sirtuins impairs their protective roles against oxidative damage and inflammation, accelerating lung aging and disease advancement (Chen et al. 2018).

### Telomere attrition

Telomere is a part of a chromosome which prevents the tangling of the chromosome during DNA replication. When cell divides, telomere gets slightly shorter each time and this process lasts on the shortening of telomere at a level that a cell cannot divide more and death of cell occurs (Chakravarti et al. 2021). The main reason for telomere shortening is the oxidative stress and incomplete DNA replication (Victorelli and Passos 2017). As time goes, this shortening of telomere gets elevated and reach a critical length. At this point, apoptosis is triggered and contributes to cellular senescence or ageing process and potentially impacting overall cellular function (Shammas 2011).

### Epigenetic alterations

In Epigenetic alterations, DNA methylation, involvement of non-coding RNAs, and modifications to histones plays an important role in senescence associated with COPD (Avci et al. 2022; Hikichi et al. 2019; Bure et al. 2022). In COPD patient, hypermethylation of DNA occurs which affects tumor suppressor genes (p16INK4a and p14ARF), this further leads to suppress the transcription and in last cell cycle arrest occurs (Al-Kaabi et al. 2014; Engeland 2018). Methylation and acetylation of histone plays an important role in regulating the chromatin structure (Miller and Grant 2012). This structure changes influence the gene expression patterns which is associated with senescence (Gharipour et al. 2021).

### Loss of proteostasis

Loss of proteostasis refers to the loss of cell's ability to regulate protein folding correctly, which is an important molecular factor in the development of COPD and also associated with cellular senescence (Balch et al. 2014). In COPD patients, certain symptoms have seen after loss of proteostasis which includes increased endoplasmic reticulum stress, impaired autophagy, and disturbed function of ubiquitin–proteasome system (Yu et al. 2022). These all changes further results in buildup of misfolded and aggregated proteins within lung cells, which causes cellular senescence (Cuanalo-Contreras et al. 2023).

### Mitochondrial dysfunction

The factors responsible for COPD like tobacco smoking, infections, inflammation, and environmental factors can disturb the protein synthesis, mitochondrial morphology, and lead to mitochondrial dysfunction (Dua et al. 2021). This dysfunction of mitochondria causes disturbances in regulation of calcium, affect cytosolic components, increase oxidative stress, and affect overall metabolic activities (Chellappan et al. 2022). All disturbed factors has been identified as a significant factor contributing to cellular senescence (Antunes et al. 2021). The disturbance in mitochondrial function is mainly characterized by increased ROS level within impaired mitochondria (Shen et al. 2022; Zorov et al. 2014). This increased levels of ROS can cause damage to mitochondrial DNA (mtDNA) which after this initiates an inflammatory reaction by activating NF-κB (nuclear factor kappa B) pathway, which can further intensify the cellular senescence (Juan et al. 2021; Shimura 2023; Vasileiou et al. 2019).

### Dysregulated nutrient-sensing

Disturbances in nutrient-sensing pathway also contributes to cellular senescence in COPD (Kang 2020). In nutrient-sensing, mTOR (mechanistic target of rapamycin) plays an important role for integrating all signals related to energy and nutrients (Hu et al. 2016). The dysregulation or imbalance of this mTOR pathway results into disruption of mitochondrial functions which further exacerbating the cellular senescence via different pathways, described in below image (Sun et al. 2023; Miwa et al. 2022).

### Stem cell exhaustion

Stem cell disruption is an important cause for COPD and cellular senescence at the molecular level (Oh et al. 2014). In COPD patients, due to high oxidative stress and continuous DNA harm, the cells like epithelial progenitor cell and mesenchymal stem cell loss its ability to regenerate itself, which ultimately cause cellular senescence (Sin 2010; Sun et al. 2018).

**Fig. 1:**
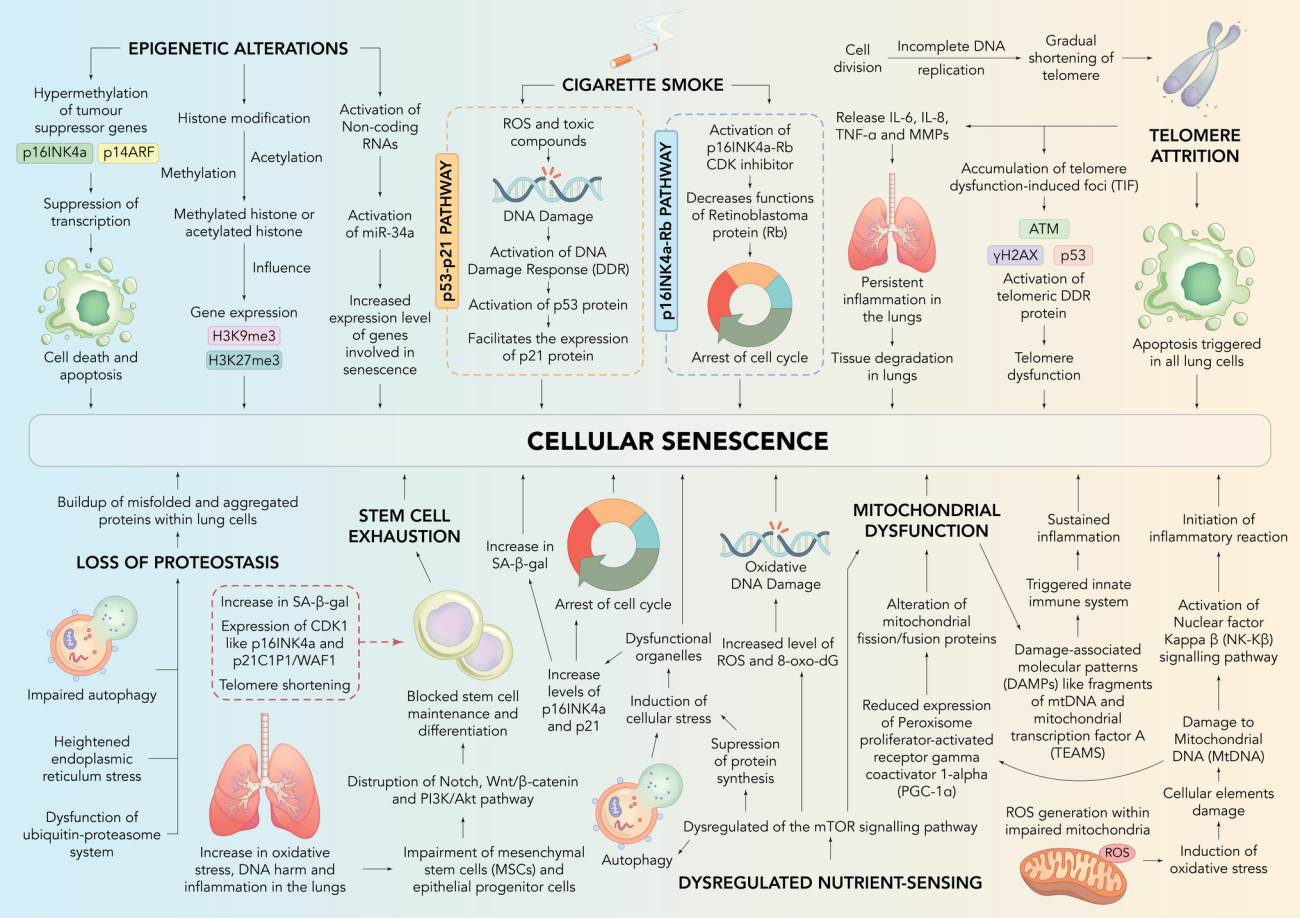
Illustration showing different molecular mechanisms contributing to cellular senescence in COPD

A dramatical representation of all molecular mechanisms involved in cellular senescence in COPD is given in Fig. 1.

## Current treatment to mitigate cellular aging in COPD and its disadvantages

Current treatments to mitigate cellular ageing in COPD include the use of repurposed drug therapies, though each comes with distinct disadvantages (Jessamine et al. 2024). Resveratrol, a plant-derived polyphenol known for its antioxidant and anti-inflammatory effects, has demonstrated potential in lowering oxidative stress and inflammation in individuals with COPD. Despite several potential benefits of Resveratrol, its efficacy in clinical settings is a big challenge due to the limited bioavailability and clinical data (Beijers et al. 2018). Metformin a commonly prescribed antidiabetic drug, has proven to have some anti-inflammatory and anti-aging potential. However its long-term effect and safety needs to investigated for the improvement in COPD symptoms (Plowman et al. 2024). Similarly, melatonin also proven to have some antioxidant and anti-inflammatory effects in COPD patients. However, its ideal dosage and mechanism need to standardized to establish its effectiveness (Wang and Gao 2021). All these treatment collectively have the potential to mitigate cellular ageing in COPD, but there are several key elements that are needed to validated to prove their potential (Singh et al. 2023).

## Nano-based drug delivery systems for mitigation of cellular aging in COPD

Nano-based drug delivery is the novel method for delivery therapeutic agents to treatment COPD. These novel systems have the potential in mitigating cellular ageing in COPD. It also offer sustained drug release, targeted drug deposition, biodegradation protection, and reduced dosing frequency (Saxena et al. 2022). Due to the distinct properties such as small size, high stability, and physicochemical properties, nanocarrier can enclose antioxidant and anti-inflammatory substances, which further can target and reduce oxidative stress in COPD (Paudel et al. 2022). These systems can also effectively deliver the genetic materials including DNA, siRNA or microRNA, that can alter the gene expression (Passi et al. 2020). This can actively target the specific genetic pathways that are involved in the pathogenesis of COPD, which can potentially reverse or slowdown the process the cell aging (Pontes and Grenha 2020). Using nanoparticles as autophagy modulator can offer several advantages such as low systemic toxicity, high drug delivery efficiency and reduction in drug resistance (Hoffmann et al. 2020). Autophagy is a intercellular pathway that is degrades and recycles long-lived proteins and cytoplasmic organelles (Din et al. 2017). Targeting mitochondria using nanocarrier entrapped protective agents can help in maintain its function and delaying cellular ageing (Suk et al. 2016). Nanocarriers can also improve immune responses and reduce chronic inflammation, which is generally responsible for cellular aging in COPD (Kia et al. 2023).

## Applications of nano-based drug delivery systems for mitigation of cellular ageing in COPD

### Liquid crystalline nanoparticles (LCNs)

LCNs are a types of nano-based drug delivery systems that have ordered structures, like that of liquid crystals with properties of nanoscale particles. Recently these nanocarriers have gain attention form researchers as these systems bridge nanotechnology and liquid crystals science (Chan et al. 2021). Several studies have proven the potential of LCNs loaded with antioxidant and anti-inflammatory compounds.

**Fig. 2:**
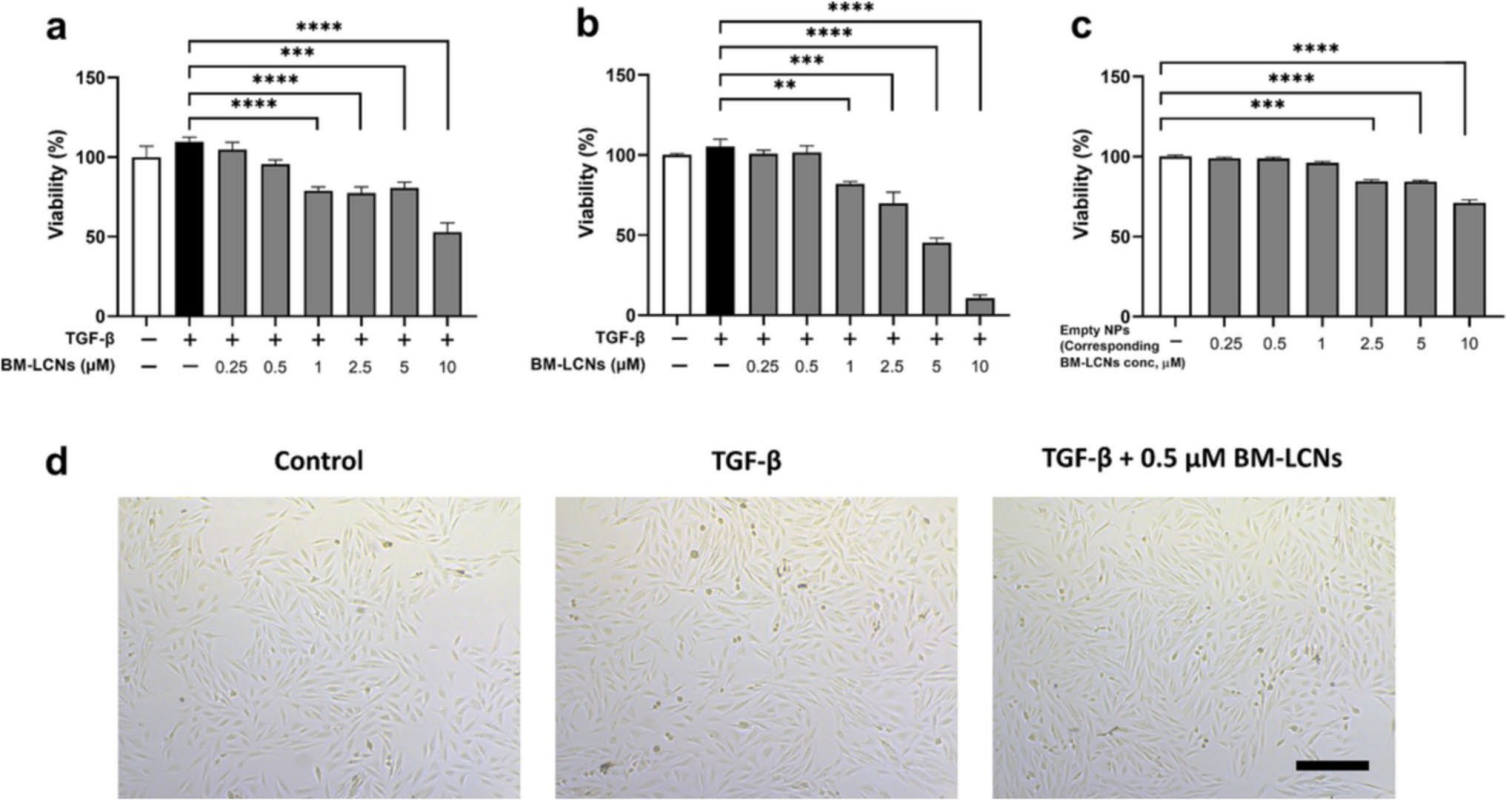
BM-LCNs treatment on cell viability and morphology of TGF-β-induced BEAS-2B cells. **a** showed MTT assay and **b** showed Trypan Blue assay, both assessed for cell viability after treated with BM-LCNs. **c** Blank-LCNs were employed as controls. **d** Cell morphology with TGF-β alone and TGF-β + BM-LCNs. ‘Results are presented as mean ± SEM (*n* = 3), with significant differences noted (**p < 0.01; ***p < 0.001; ****p < 0.0001, one-way ANOVA). *Scale bar* = 300 μm’ (De Rubis et al. 2023a, b) (used with permission from Elsevier)

Berberine, a common quinoline alkaloid derived from Chinese medicinal herbs, is primarily obtained from *Coptis chinensis*. Studies indicate that berberine exhibits anti-inflammatory, antioxidant, lipid-lowering, antiarrhythmic, blood glucose-reducing, and liver-protective properties. De Rubis and colleagues explored the effectiveness of berberine in addressing airway remodeling associated with chronic respiratory conditions such as COPD and asthma. Airway remodeling refers to the alterations in the quantity or arrangement of the cellular and molecular components within the airway wall. Airway remodeling is linked to the underlying mechanisms of airway conditions like asthma and Chronic Obstructive Pulmonary Disease. The key characteristics of the disease include abnormal epithelial repair, increased extracellular matrix (ECM) accumulation, epithelial to mesenchymal transition (EMT), and airway blockage. The poor permeability of berberine was addressed by encapsulating it in monoolein-based liquid crystalline nanoparticles (BM-LCNs). These BM-LCNs notably suppressed TGF-β-induced migration of human bronchial epithelial cells (BEAS-2B) and decreased the expression of several key pro-remodeling proteins such as endoglin, thrombospondin-1, basic fibroblast growth factor, vascular endothelial growth factor, and myeloperoxidase. In Addition, BM-LCNs counteracted the TGF-β-mediated reduction in cystatin C levels and restored nitric oxide (NO) secretion to normal levels in vitro. These findings demonstrate that BM-LCNs are effective against the airway remodelling features induced by TGF-β, and further support this could be a novel therapeutic strategy for CRDs utilizing berberine delivered via nanoparticle delivery systems (De Rubis et al. 2023a, b). Some important outcome of this study is given in Fig. 2.

**Fig. 3:**
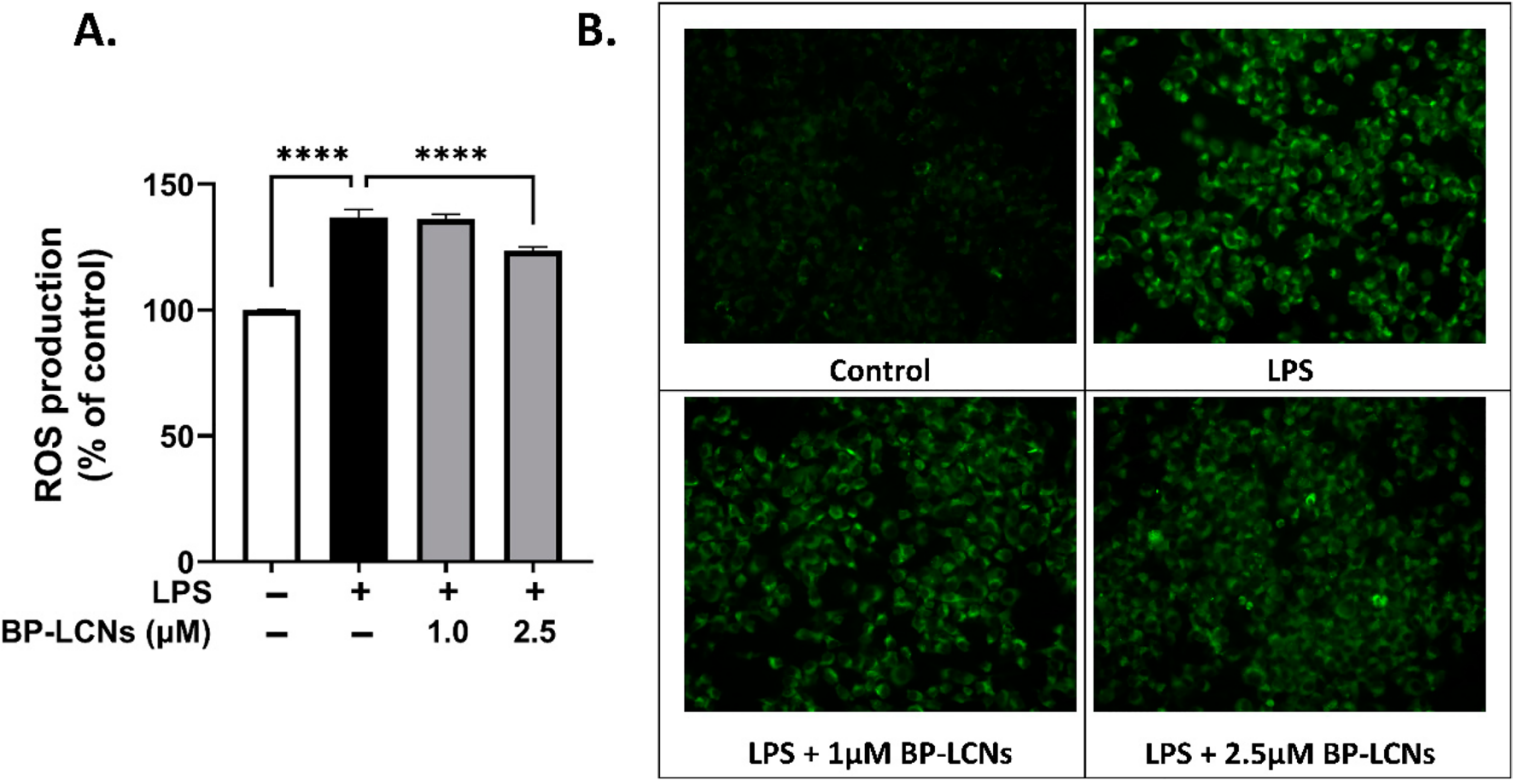
The BP-LCNs potential on LPS-induced ROS production in RAW 264.7 cells are displayed. **A** ROS production was recorded using fluorescence plate reader and **B** fluorescence microscopy. ‘Results are presented as Mean ± SEM of three independent experiments (****p < 0.0001)’ (Alnuqaydan et al. 2022). (Copyright © 2022 the Author(s). Published with MDPI and no special permission is required)

Alnuqaydan and co-authors also prepared the Berberine-loaded Phytantriol-based LCNs (BP-LCNs) to solve to issue of poor solubility and bioavailability with berberine. Their research found that BP-LCNs exhibited potent antioxidant and anti-inflammatory activities (which is directly associated with cellular senescence) in LPS-induced mouse macrophages at a less concentration of 2.5 µM (Fig. 3). This research emphasizes the potential of BP-LCNs as a therapeutic agent for CRDs, showcasing their enhanced efficacy and reduced toxicity compared to conventional berberine administration (Alnuqaydan et al. 2022).

Mehta and her colleagues developed Rutin-loaded Liquid Crystalline Nanoparticles (RT-LCNs) for their inhibitory effects on oxidative stress using BEAS-2B cells. The researchers observed that RT-LCNs effectively suppressed the expression of Nox2B and Nox4 genes, which are implicated in oxidative stress induction (Mehta et al. 2021).

Paudel and colleagues developed Zerumbone-loaded Liquid Crystalline Nanoparticles (ZER LCNs) to protect against inflammation, oxidative stress, and cellular senescence caused by cigarette smoke extract (CSE) in an in vitro model. They tested the antioxidative, anti-inflammatory, and anti-senescence effects of the ZER LCNs using CSE-treated mouse macrophage (RAW264.7) and human basal epithelial (BCi NS1.1) cell lines. Through this evaluation the team, demonstrated that prepared ZER LCNs has significantly reduced pro-inflammatory markers such as II-6, Tnf-∝ and II-1β, while also suppressing the production of nitric oxides in macrophages. ZER LCNs also reduced the ROS levels and modulate the gene (GPX2 and GCLC) in human basal epithelial cell lines, which lead to inhibition of oxidative stress. In Addition, it also exhibited anti-senescence activity in human basal epithelial cell lines, which significantly reduced the expression of CDKN1A, CDKN2A and SIRT1. This study finding highlights the potential of ZER LCNs as multitargeted therapy for treatment of COPD and asthma (Paudel et al. 2023).

### Nanoemulsions

Nanoemulsion is a kinetically stable, thermodynamically unstable colloidal dispersion of two immiscible liquids. They are made by e.g., high shear emulsification stabilized by surfactants and/or co-surfactants. Microemulsions on the other side, have a similar goblet size, however, they are self-emulsifying systems and do not require high energy processing. It can be either water-in-oil (w/o) or oil-in-water (o/w) systems (Malik et al. 2023). It is a widely used drug delivery system in food, pharmaceutical, and cosmetic industry. This nano system is a promising frontier in the treatment of COPD (Devkota et al. 2022).

**Fig. 4:**
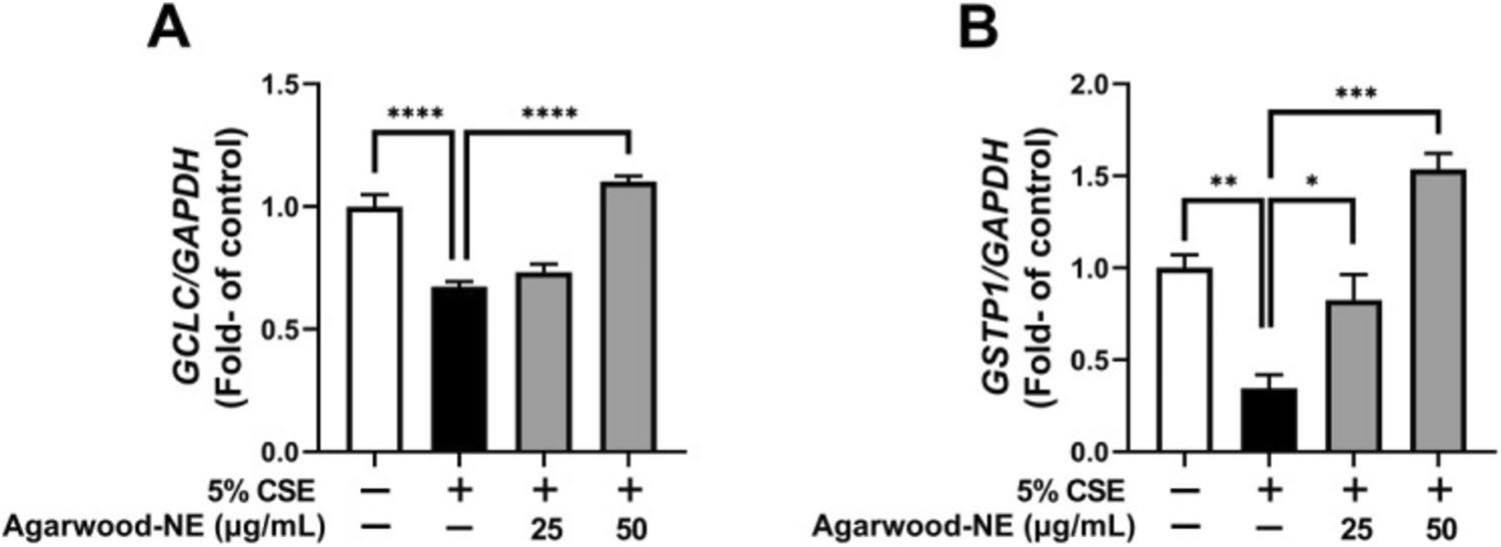
The effect of Agarwood-NE on CSE-inhibited transcription of antioxidant genes in BCi-NS1.1 cells are shown. **A** The mRNA levels of GCLC and **B** GSTP1 were measured, with ‘results expressed as mean ± SEM (*n* = 3–4). Statistical significance is indicated as follows: *p < 0.05; **p < 0.01; ***p < 0.001; ****p < 0.0001’ (De Rubis et al. 2023a) (Copyright © 2023 the Author(s) Published with MDPI and no special permission is required)

Rubis and colleagues examined the ability of agarwood oil nanoemulsion (Agarwood-NE) to alleviate oxidative stress and inflammation triggered by cigarette smoke. Their findings demonstrated that Agarwood-NE effectively reduced cigarette smoke extract-induced pro-inflammatory responses in BCi-NS1.1 airway epithelial cells by lowering the levels of pro-inflammatory cytokines including IL-1α, IL-1β, IL-8, and GDF-15, while simultaneously enhancing the expression of anti-inflammatory mediators such as IL-10, IL-18BP, TFF3, GH, VDBP, relaxin-2, IFN-γ, and PDGF. Agarwood oil nanoemulsion also upregulated GCLC, GSTP1, which are antioxidant genes (Fig. 4). These findings highlight the effectiveness of agarwood oil nanoemulsion for COPD (De Rubis et al. 2023a).

**Fig. 5:**
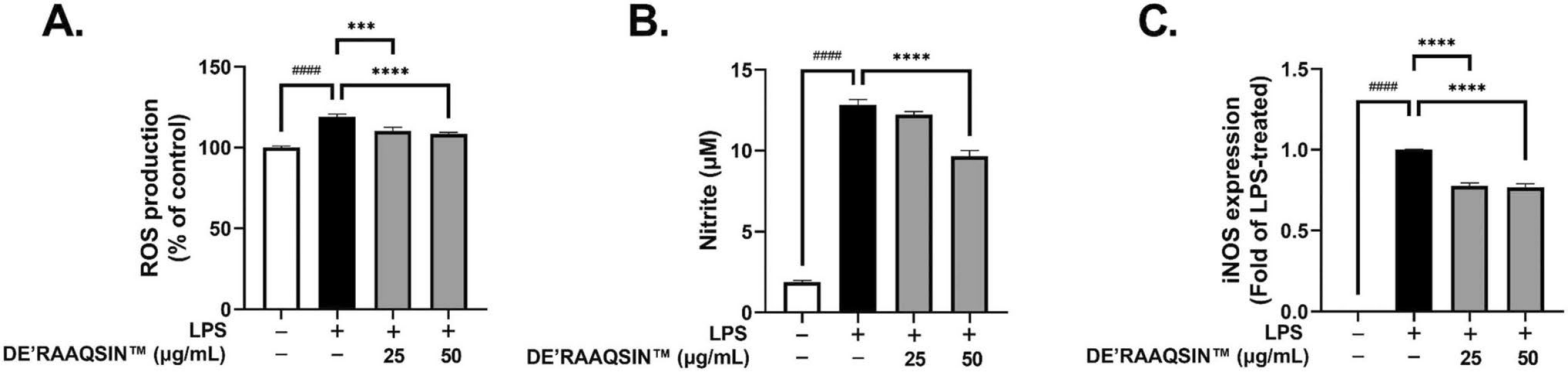
Effect of *DE’RAAQSIN* on LPS-induced ROS and NO generation and iNOS expression in RAW264.7 cells are shown. **A** Cells treated with LPS and *DE’RAAQSIN*, and then analyzed for ROS levels, **B** NO production, and **C** iNOS mRNA levels. ‘Data are presented as Mean ± SEM (*n* = 3, ***p < 0.001 vs. LPS-treated, ****p < 0.0001 vs. LPS-treated, ^####^*p* < 0.0001 vs. control, One-Way ANOVA)’ (Malik et al. 2023) (used with permission from Elsevier)

Malik and her team investigated ‘*DE'RAAQSIN’*, an agarwood oil-based nanoemulsion, for its potential in addressing inflammation and oxidative stress in chronic respiratory diseases like asthma and COPD. Their findings revealed the efficacy of *DE’RAAQSIN* on RAW264.7 cells in mouse and found the decreased level of NO and ROS in cell as well as the over-expression of pro-inflammatory genes. The results indicated that *DE'RAAQSIN* has potent anti-inflammatory and antioxidant activity (Fig. 5), suggesting its potential as an alternative for managing inflammatory disorders and warranting further investigation into its effectiveness (Malik et al. 2023).

### Solid lipid nanoparticles (SLNs)

Solid lipid nanoparticles (SLNs) are emerging as a promising drug delivery system, particularly for pulmonary applications. Their design consists of a solid lipid core surrounded by surfactants, which provides several advantages for delivering therapeutics to the lungs (Sivadasan et al. 2023).

**Fig. 6:**
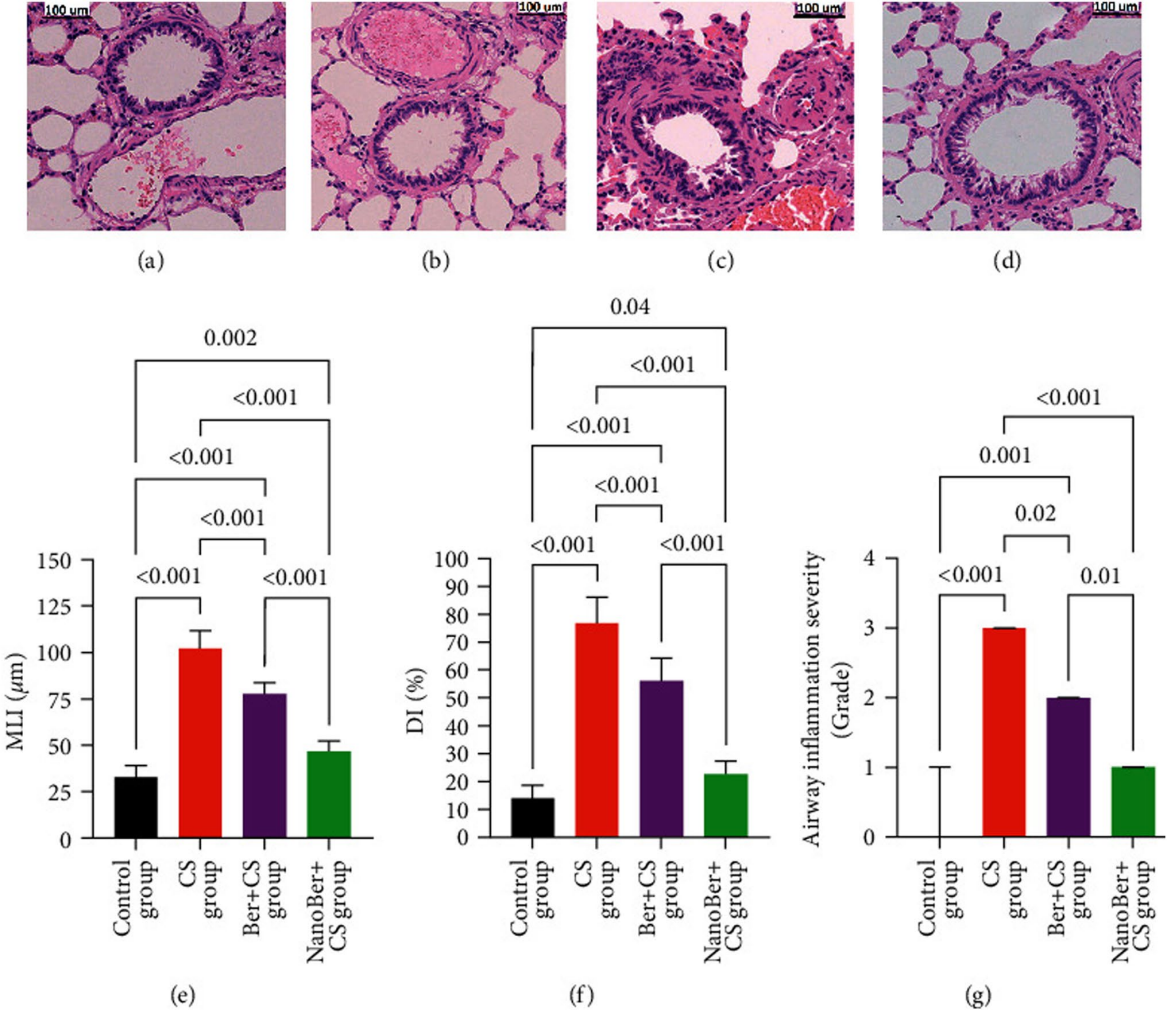
Histologic analysis of lung tissue showing the effects of Ber-loaded SLN-chitosan nanoparticles and pure berberine suspension on CS-induced alterations are shown. Images include **a** control, **b** CS group, **c** Ber + CS group, and **d** Nano-Ber + CS group ‘(original magnification × 200, scale bar 100 µm)’. **e** Measurements of MLI and **f** DI, indicated changes in airway space and structure. **g** The severity of airway inflammation is graded on a 0–3 scale. ‘Data are expressed as mean ± SD (*n* = 10), with statistical significance set at *p* < 0.05’ (Liu et al. 2022) (Copyright © 2022 the Author(s) Published with Open Access and no special permission is required)

Liu and the team investigated the therapeutic potential of Berberine encapsulated in Chitosan-coated SLNs (Ber-SLE-chitosan) for treating COPD. Results showed that the nanoparticles had a nano-range particle size, a controlled release profile and was highly stabile. When administered to COPD rats, the Ber-loaded nanoparticles significantly reduced lung inflammation, levels of cytokines, and activities of Superoxide Dismutase (SOD) and Myeloperoxidase (MPO) enzymes in Bronchoalveolar Lavage Fluid (BALF), compared to pure berberine (Fig. 6) (Liu et al. 2022).

Similarly, Castellani et al. explored the potential of grape-derived phenolic compounds, recognized for their antioxidant, anti-inflammatory, anti-aging and anti-cancer properties. To address the low water solubility of natural anti-oxidants like proanthocyanidins, the researchers developed a SLN drug delivery system by encapsulating Grape Seed Extract (GSE). In mice, SLNs were detectable in the lungs for up to 6 days. GSE-loaded SLNs, with a mean diameter of 243 nm, were negatively charged, and stable at 37 °C in simulated lung fluid till 48 h. These SLNs significantly reduced production of ROS when applied 24 to 72 h prior to hydrogen peroxide stimulation, with a more pronounced effect at 48 and 72 h compared to free GSE (Castellani et al. 2018).

### Liposomes

Liposomal drug delivery systems have garnered significant interest given their potential in the treatment of COPD and other respiratory illnesses, as liposomes can encapsulate therapeutic substances such as antioxidants and anti-inflammatory agents (Xu et al. 2020). Liposomes are spherical structures composed of phospholipid bilayers, capable of encapsulating both hydrophilic and hydrophobic drugs. Their biodegradability and the biocompatibility reduce local irritation in lung tissues, enhancing the effectiveness of treatment (Nakhaei et al. 2021).

Research on liposomal dry powders for pulmonary administration of NAC, a potent antioxidant, was conducted by Ouique and co-authors. Characterization of the resulting powders for various parameters and importantly, the liposomal encapsulation of NAC preserved the antioxidant activity of NAC after drying and showed superior in vitro antioxidant activity of the encapsulated formulations compared to non-encapsulated formulations. This encapsulation not only decreased lung permeability index but also decreased inflammatory mediators in models of acute oxidant induced lung injury suggesting it as a treatment for oxidative stress related pulmonary diseases (Ourique et al. 2014).

**Fig. 7:**
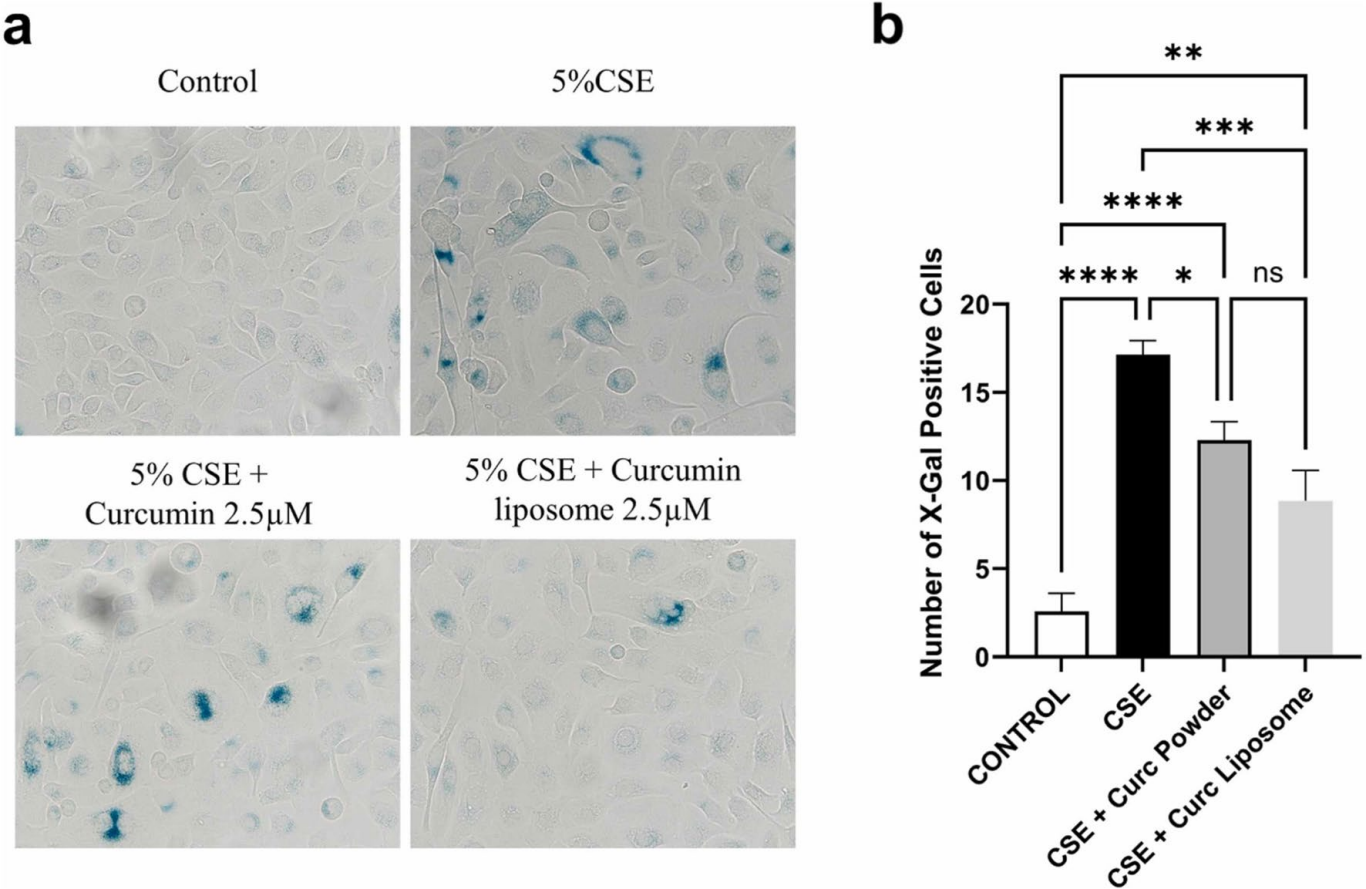
The impact of curcumin powder and liposomal curcumin on reducing senescence in BciNS1.1 cells induced by 5% CSE are shown. **a** Microscopic images at 40× magnification (*n* = 7). **b** Quantified results. ‘Statistical significance: *ns* not significant, ^####^*p* < 0.0001 vs. control, ****p < 0.0001, ***p < 0.001, **p < 0.01, *p < 0.05 vs. 5% CSE’ (Kokkinis et al. 2024) (Copyright © 2024 the Author(s) Published with Open Access and no special permission is required)

Kokkinis and authors developed curcumin-loaded liposome (termed PlexoZome®) to improve its bioavailability and therapeutic efficacy. The study utilized minimally immortalized cigarette smoke exposed human bronchial epithelial cells. At a concentration of 5 µM, PlexoZome® curcumin demonstrated significant anti-senescent effects, evidenced by a reduction in senescence markers such as p16 and p21, as well as inflammatory proteins like GMCSF and EGF (Fig. 7). This highlighted the effectiveness of liposomal technology in enhancing the therapeutic efficacy of curcumin for managing COPD, suggesting a novel approach to respiratory disease treatment (Kokkinis et al. 2024).

### Exosomes

Exosomes are extracellular vesicles measuring between 30 and 150 nm in diameter, containing molecular components derived from their originating cells. They are secreted by various cell types, including immune and tumor cells, and are crucial for intercellular communication (Kalluri and LeBleu 2020). Several studies prove the role of exosomes in the pathogenesis of COPD and its potential as therapeutic targets and biomarkers.

A study investigating the therapeutic potential of MSC and MSC derived exosomes (EXO) to stop the inflammation and mitochondrial dysfunction in COPD caused cigarette smoke, was conducted by Maremanda and the team. To study the therapeutic effect of MSC and EXO, mitochondrial reporter mice (mito-QC and mt-Keima) were used. Through this study it is observed that inflammatory cellular infiltration in the lungs of mt-Keima was increased with the exposure to Cigarette smoke. Treatment with the combined therapy (MSC + EXO) yield better results in comparison to individual treatments. Exposure to CS resulted in an increase in the mitochondrial fission protein DRP1 and other mediators of the DAMPs pathway, including S100A4, S100A8, HMGB1, RAGE, and AGE. In Addition, treatment with MSC combined with EXO elevated the gene expression of fusion-related genes mfn1, mfn2, and opa1. This study indicates that the MSC + EXO combination therapy may counteract the early effects induced by CS exposure due to its anti-inflammatory properties and mitochondrial transfer mechanisms (Maremanda et al. 2019).

### Dendrimers

Dendrimers are globular, monodisperse macromolecules characterized by unique three-dimensional polymeric structures, where all bonds radiate outward from a central core, and each repeating unit provides a branching point (Abbasi et al. 2014). Several studies have highlighted their potential as the carriers for therapeutic agents which are aimed at addressing cellular aging and inflammation in COPD.

Zhong and colleagues investigated how the route of administration and PEGylation alter the biodistribution of generation 3, PAMAM dendrimers (G3NH2) for pulmonary drug delivery. Pharmacokinetic profiles indicate that the chemistry of the dendrimers has no effect on the time required to reach their peak concentration in systemic circulation after pulmonary delivery. Nevertheless, pulmonary absorption and the peak plasma concentration are increased by high density of surface modification with PEG. Approximately 20% of lung endothelial cells internalize G3NH2-24PEG1000 following pulmonary administration, whereas only 6% do so for G3NH2. In contrast, lung epithelial cells take up G3NH2 more efficiently (35%) compared to its PEGylated form (24%). These findings demonstrate that both the pulmonary delivery route and dendrimer surface chemistry can be leveraged to passively direct drug carriers to specific tissues and cell types, providing valuable insights for designing dendrimer-based drug delivery systems aimed at treating lung diseases and systemic conditions (Zhong et al. 2016).

### Polymeric nanoparticles (NPs)

Polymeric micelle is a bilayer structure, one with an inner core for hydrophobic components and the other with an outer shell for hydrophilic components. The outer shell is also known as the corona (Beach et al. 2024). Several research highlights the potential of NPs in reducing ROS production and mitochondrial dysfunction in COPD (Huang et al. 2021).

To deliver microRNA (miR-146a) to inhibit the IRAK1 protein expression in COPD, Mohamed and colleagues developed cationic polyglycerol adipate-co-ω-pentadecalactone (PGA-co-PDL) nanoparticles incorporating dioleoyltrimethylammoniumpropane (DOTAP). The NPs were prepared by an oil-in-water single emulsion solvent evaporation method and had a size of 244.80 ± 4.40 nm and a zeta potential of +14.8 ± 0.26 mV at 15% DOTAP, and miR-146a adsorption was 36.25 ± 0.35 µg per 10-mg NPs after 24 h. Over 75% of A549 cells were viable in vitro at 0.312 mg/ml and miR-146a was found to be released over 24 h with sustained release (77%). Confocal and fluorescence microscopy demonstrated that NP were internalized, miR-146a-NPs at 0.321–0.625 mg/ml decreased expression of IRAK1 by about 40% and reduced IL8 promoter activity, thus demonstrating retained biologic activity. These findings indicate that the modulating inflammatory pathways using PGA-co-PDL-DOTAP NPs as the delivery platform may be used to deliver miR-146a based COPD therapeutics (Mohamed et al. 2019a, b).

Rubis and colleagues explored the therapeutic potential of 18-β-glycyrrhetinic acid (18-β-gly), a licorice-derived phytoceutical known for its anti-inflammatory, antioxidant, and antiviral properties, which is limited in clinical use due to its poor solubility. In their study, they encapsulated 18-β-gly within poly lactic-co-glycolic acid (PLGA) nanoparticles to improve its delivery and efficacy in addressing the impaired antiviral response seen in cigarette smokers and COPD patients It is demonstrated that the partial restoration of these chemokine expression by treatment with 18-β-gly-PLGA nanoparticles indicates that the nanoparticle-based delivery system may attenuate the detrimental effects of cigarette smoke on antiviral defenses. The results show promise of 18-β-gly-PLGA nanoparticles as a novel therapeutic strategy to enhance the antiviral immunity in such vulnerable populations (De Rubis et al. 2024).

### Nanocomposite microparticles (NMs)

Nanocomposite microparticles are type of drug delivery systems that combine the unique characteristics of nanomaterials and microparticle systems. These are microparticles which incorporate nanocomposite structures (Abbasi et al. 2023). These drug delivery systems are designed to improve COPD treatment by enabling targeted and sustained drug delivery directly to the lungs.

Mohamed and the team formulated nanocomposite microparticles consisting of miRNA (miR-146a0, as novel inhalation therapy for COPD. The team developed NMs by formulating PGA-co-PDL nanoparticles as drug powder inhalation with L-leucine and mannitol as excipients by varying their ratios. Among all the formulation developed, formulation with 25:75 (L-leucine: mannitol) has 86.0% yield, low moisture content (2.02%), with a high fine particle fraction and emitted dose and mass median aerodynamic diameter which is suitable for long deposition (Mohamed et al. 2019b).

## Nanotubes

These are nanosized cylindrical structure with a hollow core that are used as carriers to deliver drugs, genes, and other therapeutics agents to the target site with the body. These delivery systems are being explored for their potential for targeted delivery, enhanced bioavailability, and reduced side effects in comparison to conventional treatments.

**Fig. 8:**
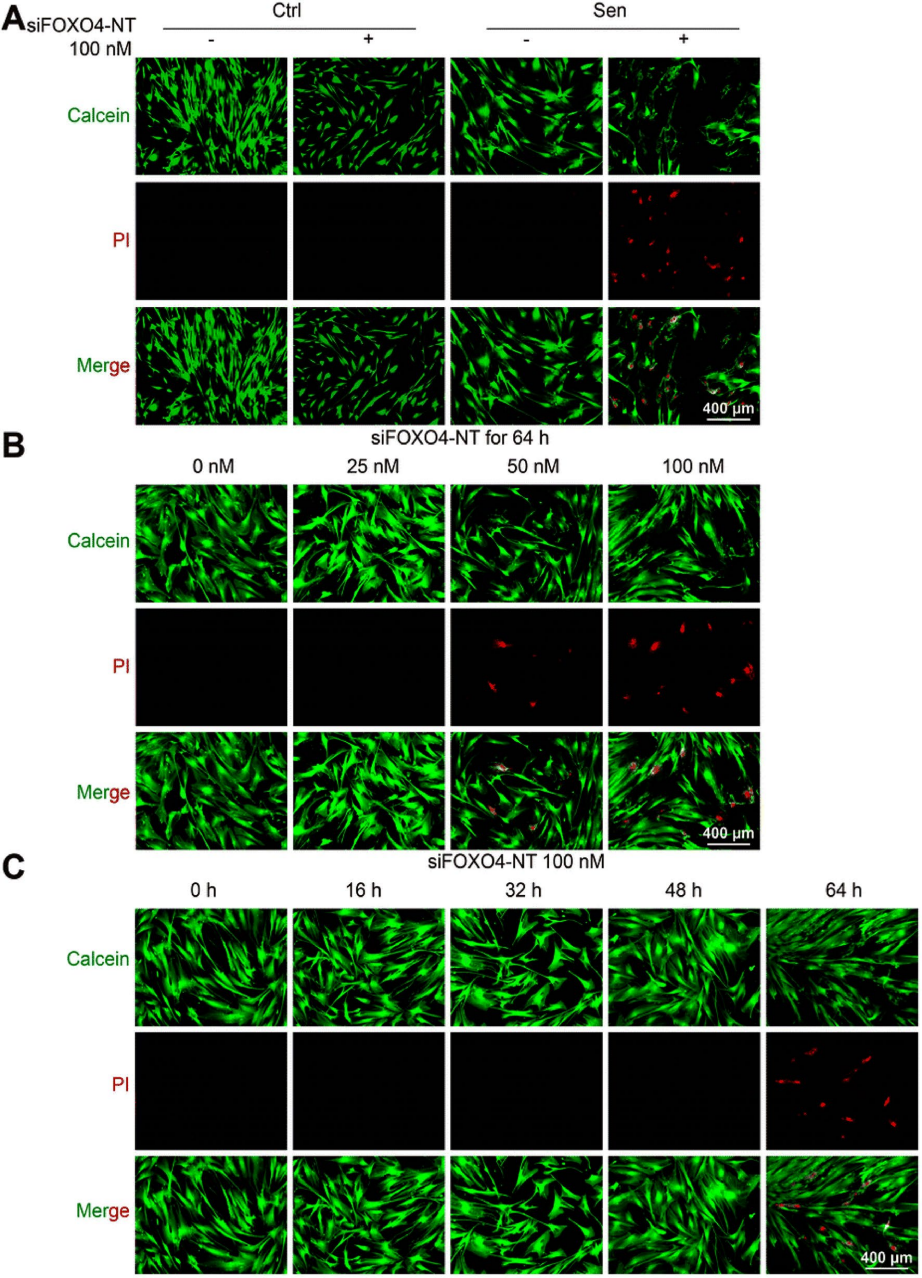
Selective elimination of CSE-induced senescence by siFOXO4-NT in HFL-1 cells. **A** normal and senescent HFL-1 cells under calcein or PI stain in the presence or absence of siFOXO4-NT. **B** apoptotic cell (*red*) and living cells (*green*). *Scale* = *400 μm*. and **C** Senescent cells under calcein/PI staining with siFOXO4-NT (Han et al. 2023) (Copyright © 2023 the Author(s) Published with Open Access and no special permission is required)

Han and colleagues investigated the relationship between cellular senescence and the pathogenesis of COPD and the possibility of senolytic drugs in the treatment of this condition. In vitro and in vivo, they found that cigarette smoke-induced senescent lung fibroblasts had elevated FOXO4 expression. To target these senescent cells, the researchers developed self-assembled DNA nanotubes (siFOXO4-NT) made from single stranded FOXO4 siRNA (siFOXO4-NT). These nanotubes had been designed to target FOXO4 in senescent fibroblasts. The research showed that siFOXO4-NT could effectively enter human lung fibroblasts (HFL-1 cells) in a concentration and time dependent manner, reducing FOXO4 levels in vitro. Notably, siFOXO4-NT selectively cleared senescent HFL-1 cells by decreasing the expression of BCLXL and altering the BCL2/BAX ratio, which were elevated in CSE-induced senescent cells (Fig. 8) (Han et al. 2023).

## Patents on nanoformulations for the attenuation of COPD

Table 1 enlists the most recent patents filed on nanotechnology-based advanced formulations for COPD.

**Table 1:** List of patents on nanoformulations targeting COPD

S.no.	Publication number	Nanoformulation	Title	Published year	Authors	Reference
1.	CN115227682A	Solid lipid nanoparticle	‘Preparation method of powder inhalation for targeted small-airway sustained-release delivery of COPD (Chronic Obstructive Pulmonary Disease) therapeutic drug’	2022	‘Li Nan, Li Xu’	https://patents.google.com/patent/CN115227682A/en?oq=CN115227682A
2.	US2021137845A1	Nanoparticle	‘Methods and compositions for treating respiratory disease’	2021	‘Gale Smith, Ye Liu, Jing-Hui Tian, Michael J. Massare, Sarathi boddapati, Gregory Glenn, Louis Fries, Iksung CHO’	https://patents.google.com/patent/US20210137845A1/en?oq=US2021137845A1
3.	WO2022023456A1	Nano embedded microparticles	‘Pharmaceutical compositions comprising nano embedded microparticles and methods of use’	2022	‘Ida Christina Hovdal, Sandra Gracin, Annica Maarit Jarke, Mikael Johan Alvin Brülls, Noemi Gaglianone, Ankur Ajmera’	https://patents.google.com/patent/WO2022023456A1/en?oq=+WO2022023456A1
4.	CN105310986A	Nanoparticles	‘Olodaterol lung targeting nanoparticle and its preparation method’	2016	‘Diao Yuanyuan, Zhang Ting’	https://patents.google.com/patent/CN105310986A/en?oq=+CN105310986A +
5.	CN106389387A	Nanoparticles	‘Roflumilast nanoparticle, and preparation and preparation method thereof’	2017	‘Lei Linfang, Wang Xin’	https://patents.google.com/patent/CN106389387A/en?oq=+CN106389387A
6.	CN117731609A	Liposome suspension	‘Inhalation tiotropium bromide liposome suspension, and preparation method and application thereof’	2024	‘Sun Zhou, Li Jing, Han Yun, Zhang Mingyue’	https://patents.google.com/patent/CN117731609A/en?oq=CN117731609A

## Limitation of current research and future prospect

Nanomedicine holds significant promise for treating COPD by enabling targeted drug delivery, sustained release, and improved bioavailability, particularly for addressing key pathologic features such as oxidative stress and cellular senescence. However, its clinical and commercial translation remains limited due to several challenges. Ensuring nanomedicine biocompatibility and safety is critical, as inhaled nanotherapeutics may cause lung damage or systemic side effects in the sensitive pulmonary environment of COPD patients. In addition, overcoming airway defense mechanisms like mucus barriers and remodeled airways without triggering immune responses complicates effective nanoparticle delivery. Current antioxidant therapies targeting oxidative stress and senescence show limited clinical success due to poor absorption and short half-lives, but nanoparticle-based delivery systems could enhance their bioavailability and targeting, although this remains largely experimental. The lack of robust, predictive experimental models that closely mimic human COPD pathophysiology-including oxidative stress and senescence-further restricts translational research. Manufacturing challenges, regulatory hurdles, and the complexity of producing consistent, scalable inhalable nanoformulations also impede clinical progress. Future research should prioritize developing multifunctional, biodegradable nanoparticles that combine targeted delivery with antioxidant and anti-senescence effects to maximize therapeutic benefits while minimizing toxicity. Employing advanced models such as 3D lung organoids, humanized animals, and ex vivo lung perfusion systems can better replicate COPD’s complex biology for more predictive testing. In addition, drug repurposing strategies using nanoparticles to deliver existing antioxidants, anti-inflammatory agents, or senolytics may accelerate clinical translation. Addressing these challenges through innovative experimental systems and strategic drug repurposing is essential to unlock the full potential of nanomedicine for COPD management.

## Conclusion

A novel approach using nanotechnology combined with drug delivery strategies in attenuating cellular ageing in COPD has been discussed in this review. COPD is well documented to cause increased burden and a decreased quality of life. Newer and novel therapeutic agents like nanomedicines have shown tremendous promise in attenuating oxidative stress, inflammation and cellular senescence/aging in COPD in comparison with traditional strategies. These include targeted drug delivery to the lungs, increasing drug effectiveness, reducing adverse side effects, enhancing both the stability of the system and maintaining drug solubility, extending the release time, increasing the penetration of the active drug and protecting therapeutic agents. Nevertheless, the number of limitations that need to be fixed keeps mounting. These include legal limitations, concerns that relate to toxicity, adverse effects and cost effectiveness. Success with several established technologies involving nanoparticles, nanobots, nanosponges, micropolymers that can act as nanocarriers is yet to be comprehensively proved in positively altering cellular ageing in COPD. There have been promises with a significant number of treatment modalities, namely, delivery of the active drug with exosomes, and to an extent with dendrimers in the area of cellular ageing in COPD, but very few studies documenting the pharmacologic and bioavailability parameters are available to validate this claim. Detailed and in-depth molecular studies involving nano-based strategies are necessary to triangulate the findings leading to the translation of therapies for cellular ageing in COPD. Cellular senescence and COPD is an emerging area which requires more dedication and collaboration between scientific experts, namely, pulmonary clinicians, translational researchers and pharmaceutical drug manufacturers. This will surely pave the path for advanced, patient-centered, effective, and compliant treatments in pulmonary clinics.
